# Bovine Leukaemia Virus: Current Epidemiological Circumstance and Future Prospective

**DOI:** 10.3390/v13112167

**Published:** 2021-10-27

**Authors:** Marawan A. Marawan, Abdulaziz Alouffi, Suleiman El Tokhy, Sara Badawy, Ihsanullah Shirani, Ali Dawood, Aizhen Guo, Mashal M. Almutairi, Fahdah Ayed Alshammari, Abdelfattah Selim

**Affiliations:** 1 The State Key Laboratory of Agricultural Microbiology, Huazhong Agriculture University, Wuhan 430070, China; i.shirani786@yahoo.com (I.S.); ali.dawood@vet.usc.edu.eg (A.D.); 2 College of Veterinary Medicine, Huazhong Agricultural University, Wuhan 430070, China; 3 Department of Animal Medicine (Infectious Diseases), Faculty of Veterinary Medicine, Benha University, Toukh 13736, Egypt; abdelfattah.selim@fvtm.bu.edu.eg; 4 King Abdulaziz City for Science and Technology, Riyadh 12354, Saudi Arabia; asn1950r@gmail.com; 5 The Chair of Vaccines Research for Infectious Diseases, King Saud University, Riyadh 11495, Saudi Arabia; mmalmutairi@ksu.edu.sa; 6 Department of Pharmaceutical Technology, Faculty of Pharmacy, Tanta University, Tanta 31111, Egypt; Suleiman.saleh@pharm.tanta.edu.eg; 7 Department of Pathology, Faculty of Veterinary Medicine, Benha University, Toukh 13736, Egypt; drsarabadwy@gmail.com; 8 Natural Reference Laboratory of Veterinary Drug Residues (HZAU), MAO Key Laboratory for Detection of Veterinary Drug Residues Huazhong Agricultural University, Wuhan 430070, China; 9 Para-Clinic Department, Faculty of Veterinary Medicine, Jalalabad 2601, Afghanistan; 10 Infectious Diseases, Medicine Department, Faculty of Veterinary Medicine, University of Sadat City, Sadat City 32897, Egypt; 11 Hubei International Scientific and Technological Cooperation Base of Veterinary Epidemiology, Huazhong Agricultural University, Wuhan 430070, China; 12 Department of Pharmacology and Toxicology, College of Pharmacy, King Saud University, Riyadh 22334, Saudi Arabia; 13 College of Sciences and Literature Microbiology, Nothern Border University, Arar 73211, Saudi Arabia; Fahdah1402@hotmail.com

**Keywords:** bovine leukosis, genome, prevalence, pathogenesis, clinical outcomes, diagnosis, control

## Abstract

Bovine leukaemia virus (BLV) is a deltaretrovirus that is closely related to human T-cell leukaemia virus types 1 and 2 (HTLV-1 and -2). It causes enzootic bovine leukosis (EBL), which is the most important neoplastic disease in cattle. Most BLV-infected cattle are asymptomatic, which potentiates extremely high shedding rates of the virus in many cattle populations. Approximately 30% of them show persistent lymphocytosis that has various clinical outcomes; only a small proportion of animals (less than 5%) exhibit signs of EBL. BLV causes major economic losses in the cattle industry, especially in dairy farms. Direct costs are due to a decrease in animal productivity and in cow longevity; indirect costs are caused by restrictions that are placed on the import of animals and animal products from infected areas. Most European regions have implemented an efficient eradication programme, yet BLV prevalence remains high worldwide. Control of the disease is not feasible because there is no effective vaccine against it. Therefore, detection and early diagnosis of the disease are essential in order to diminish its spreading and the economic losses it causes. This review comprises an overview of bovine leukosis, which highlights the epidemiology of the disease, diagnostic tests that are used and effective control strategies.

## 1. Introduction

Bovine leukaemia virus (BLV) is a *deltaretrovirus* that belongs to the *Retroviridae* family. It is closely related to human T-cell leukaemia virus types 1 and 2 (HTLV-1 and -2) [1,2] and to simian T-cell leukaemia viruses (STLVs). Some of them are also thought to play a role in proliferative or neurological disorders of human and non-human primates [3,4]. BLV is the causative agent of enzootic bovine leucosis (EBL), also known as bovine leucosis, which is the most common neoplastic disease of dairy and beef cattle [1,5,6].

Most BLV-infected cattle (about 70%) are asymptomatic (subclinically infected). Hence, there is an extremely high shedding rate of BLV within cattle populations and its control is rendered unfeasible [7]. About 30% of BLV-infected cattle develop persistent lymphocytosis (PL) and around 1–5% may develop tumours in a form of malignant B-cell lymphosarcoma after a long period of latency (one to eight years) [1,8,9].

The immune systems of infected cattle are impaired, even during the latent stages of leukaemia, and this leads to the inability of the animals to maintain normal performance [10]. Therefore, BLV infection results in negative effects on animal health and productivity. The disease causes huge economic losses worldwide through both direct and indirect costs: directly because milk production is reduced, the disease has an extreme impact on reproduction, and some cows must be culled prematurely; and indirectly because imports are restricted of animals from BLV-infected areas [11,12,13,14,15]. For these reasons, the World Organisation for Animal Health (OIE) has listed EBL as a disease that can cause drastic impacts on international trade [16]. For instance, BLV infects more than 40% of the United States cattle population [17] and annual economic losses have been estimated at USD 525 million from milk loss alone [18,19].

Regarding the public health hazards of bovine leucosis, there is no definitive determination of how humans are infected by BLV or of its effects in infected people. BLV proviral DNA has been found in milk and meat products, which has focused attention on the possible transmission of the disease to humans via such foodstuffs [20]. Several reports show that BLV may harm humans through a possible link between BLV infection and the development of breast cancer in women, as well as other haematopoietic neoplastic diseases [21,22,23,24,25]. Comparison of data indicates a surprising geographical correlation between incidence of breast cancer and the consumption of bovine meat and milk. For example, the UK, Australia, the US, and Germany each have a high prevalence of breast cancer along with high rates of consumption of bovine meat and milk. In contrast, Japan, India, China, and Korea have low rates of consumption of these products and low prevalence of breast cancer [26,27]. Thus, prevention and control policies should be established to diminish viral prevalence and transmission rates among cattle populations and to ensure the absence of infected foodstuffs in the market [20].

BLV infection can be diagnosed either by use of serological tests (e.g., through agar gel immunodiffusion (AGID), a radioimmunoprecipitation assay (RIA) or an enzyme-linked immunosorbent assay (ELISA)) or by use of proviral DNA detection techniques (e.g., single, semi-nested, nested, or real-time polymerase chain reaction (PCR) tests), which are sensitive and specific methods [16].Vaccination would be effective to control the disease but unfortunately, to date, there is no commercially available vaccine against BLV to prevent EBL because all tested methods have produced only incomplete or transient stimulation of the host immune response [11,28,29,30]. The most potent, alternative control measure is currently based on the testing of animals and elimination of those that are positive. However, this strategy is not feasible, especially in areas of high BLV prevalence. The aim of this review is to highlight the current epidemiological situation regarding BLV infection and to provide information about some strategies that can be used to fight the disease worldwide, with special reference to some future prospective studies.

## 2. BLV Genome

BLV is a single-strand, diploid RNA virus, the complete genome of which comprises a 8714-nucleotide sequence [1,31]. It can be anatomised in the form of (long terminal repeat (5′-LTR))-*gag*-*pro*-*pol*-*env*-*px*-(long terminal repeat (3′-LTR)) [1,28,32,33]. The 5′- and 3′-LTRs contain transcriptional promoters for the action of the Tax protein and are composed of three main regions, namely, the U3, R, and U5 regions [34]. Additionally, it has been reported that BLV particle synthesis is accelerated by mutations in the genes of the LTR region and that this acceleration results in a more intense immune response in cattle [35]. The *gag* gene is highly conserved and consists of a 1178-nucleotide sequence that codes for the polypeptide precursor Pr44, which is subsequently cleaved into three major non-glycosylated proteins (p12 nucleocapsid, p24 capsid, and p15 matrix) by the action of BLV protease [36,37]. The viral protease p14 (*pro* gene) is encoded by a region located between the *gag* and *pol* genes. It is responsible for the post-translational maturation of BLV [38]. The *pol* gene encodes for reverse transcriptase and integrase enzymes that are responsible for reverse transcription and integration of the BLV proviral DNA into the host genome, which results in life-long infection [39,40].

The *env* gene is encoded by the polypeptide precursor gpr72, which is cleaved into two glycoproteins, a surface (SU) protein, gp51, and a transmembrane (TM) protein, gp30 [37]. These glycoproteins play an important role in the viral life cycle because they contain the recognition site required for viral entry and they mediate cell fusion [41]. The *env* gene shows a genetic polymorphism that may be useful in phylogenetic studies and classifications of BLV isolates, as confirmed by several studies [42]. The nucleotide sequence and amino acid composition of the *env* gene are useful genomic markers of BLV for the study of its distribution and to reveal the presence of different genotypes that correlate with geographic origin [32,43]. Therefore, at least 11 BLV genotypes have been detected through sequencing and phylogenetic analysis of the partial and full-length gp51 *env* gene. Genotype 1 is the most prevalent, and it and genotype 1 and 3 are common in the US, Japan, and Korea [44]. Genotypes 1, 2, 3, 4, 5, 6, and 9 have been found in South America (genotype 9 particularly in Bolivia). Genotypes 4, 7, and 8 are common in Russia and Eastern Europe. Genotype 10 is prevalent in China, Vietnam, Thailand, and Myanmar [33,45,46,47,48,49]. Genotype 4 was present at some countries from North America, South America, Africa, and Asia [42,50]. Genotype 11 are recorded in China [51] and G6 genotype has been found in South American countries, such as Argentina, Brazil, Peru, Paraguay, and Bolivia [48,52], as well as Asian countries such as the Philippines, Thailand [53], and India [54]. Moreover, genotype 1 was reported in Egypt [55]. Since every new genotype may show unique features of interaction with the host organism and leukaemogenesis is influenced by some genotypes, it is important to monitor the origin of new virus mutations for veterinary and animal husbandry [56,57] (Table 1).

In addition to encoding the previously mentioned essential and structural genes, BLV provirus also encodes additional accessory and regulatory or non-structural genes from the pX region of the genome, which is located between the *env* gene and the 3′-LTR. Unlike other retroviruses, BLV has an additional *tax* gene that results from alternative splicing of the pX region. This additional gene has a crucial role in BLV biology. The Tax protein acts as a trans-activator of BLV provirus transcription and is oncogenic to host cells; it causes their malignant transformation by disturbance of cellular growth and DNA repair inhibition [27]. It also exerts a severe impact on both stress and immune responses [65]. Likewise, the polymorphism of the *tax* gene is important in the determination of the output of BLV infection; A and H variants of *tax* have been found to be correlated with decreased whole-blood counts among BLV-negative animals and, thus, could be the hallmark of the asymptomatic leukosis of BLV infection [66].

**Figure 1:**
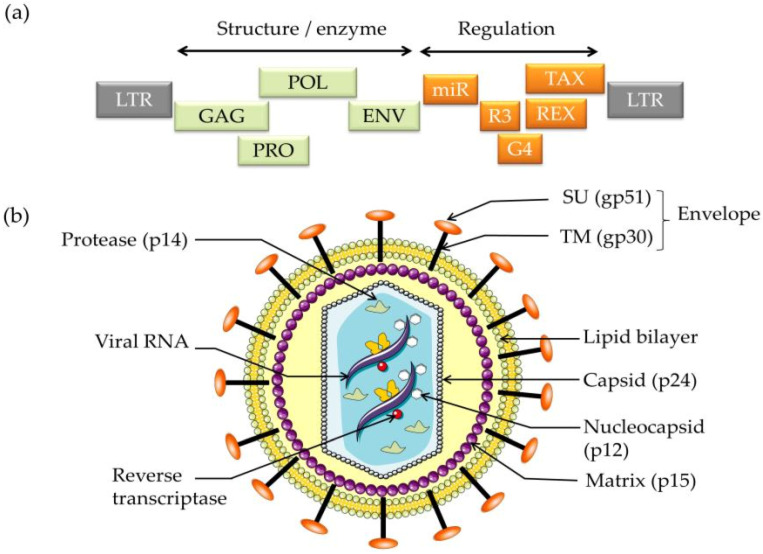
Schematic structure of (**a**) the bovine leukaemia virus (BLV) genome (**b**) the viral particle [68].

In addition to the Tax protein, the pX region also encodes for the *rex* and the less abundant R3 and G4 proteins. The Rex protein is a post-transcriptional regulator of viral expression that is required for the synthesis of structural genes and is essential for infectivity in vivo [39]. The *R3* and *G4* genes are infectious and tumourigenic BLV molecular clones that maintain high proviral load; their deletion induces loss of the leukaemogenic phenotype of BLV [67] (Figure 1).

Some researchers have reported that an abundant cluster of RNA polymerase III-transcribed microRNAs (miRNAs) is expressed even in the absence (silence) of genomic and subgenomic transcripts from the 5′-LTR region [69]. However, recently it has been shown that BLV constitutively expresses antisense transcripts from the 3′-LTR, which may be the reason for the silence of the 5′-LTR region. The BLV miRNAs are not essential for infectivity but have been shown to modify some genes that are related to other mechanisms, such as apoptosis, immunity, cell signalling, and oncogenesis. Moreover, they can be expressed in both cattle that show signs of EBL and asymptomatic leukosis-infected animals [70,71]. The transcriptional interference between antisense and mi-RNAs strongly suggests a common role in BLV regulation and represents a novel pathway for recognition of the disease [72]. The discovery of BLV antisense transcripts, together with the discovery of BLV microRNAs, has resulted in a new understanding of BLV. The BLV provirus produces a large number of viral microRNAs and expresses antisense transcripts in all malignancies studied. The presence of these transcripts in both leukaemic and non-malignant clones suggests that they play an important role in the virus’s life cycle and tumourigenic potential [72].

## 3. BLV Prevalence

BLV was first described in 1871 in Lithuania (on the south-eastern shore of the Baltic Sea) [73]. It is now known to be distributed worldwide at different prevalence rates [74]. The US has a very high BLV prevalence rate that has been stated as 40% [17]. In Japan, EBL was listed as a notifiable disease in 1998. In 2000, it was reported in 159 cattle on 157 farms, and by 2007, in 838 cattle on 677 farms [75]. In 2015, 78% of 315 dairy herds in Canada showed BLV antibodies [76]. In Argentina, 84% of dairy herds have been reported to carry the antibodies [58], in Turkey, 2.28% [77], and in Mexico, between 11% and 66% [78]. In China, a meta-analysis study was conducted to measure the prevalence of BLV during the period between 1983 and 2019. It recorded a 10% pooled BLV prevalence rate (4701 seropositive animals of 34,954 animals) [79]. BLV has also been recorded in some Middle Eastern countries [74]. In Egypt, BLV has been recorded serologically in Egyptian dairy cattle at 15.83% [80] and at 20.8%, 9%, and 0% in cattle, buffaloes, and camels, respectively.

**Figure 2:**
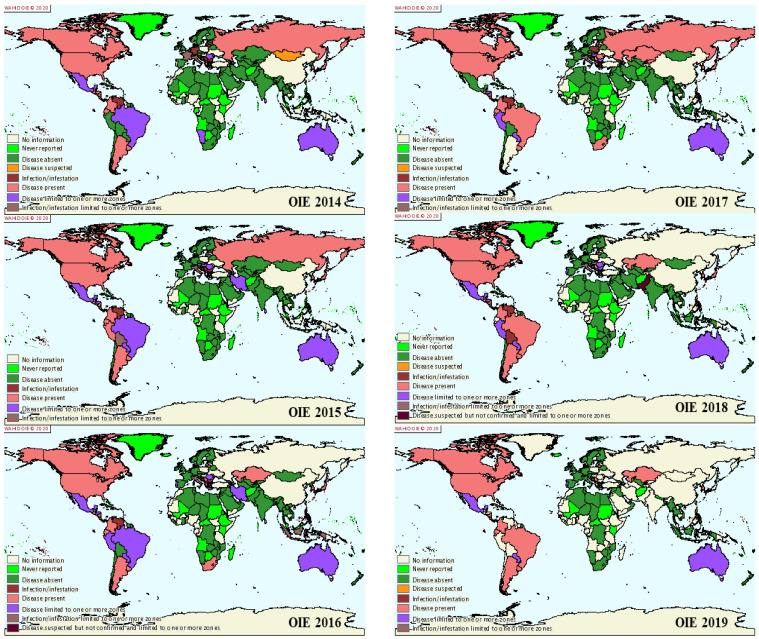
EBL world distribution map based on the last 5 years (2014–2019) [16].

In contrast, several European regions started to control the virus early in the 1960s; as a result, England, France, Germany, Spain, Belgium, Denmark, Sweden, Switzerland, Poland, and many others are officially free of BLV [81,82]. Other countries, such as Italy and Portugal, report extensive BLV-negative localities, and the infection is restricted to small areas. In Australia, the virus has been eliminated from dairy herds, but beef cattle remain infected at very low prevalence rates [7,83,84]. More than 21 countries claim to have eradicated BLV, primarily through culling or the segregation of all cattle that have been shown to be positive through ELISA tests [83,84] (Table 2 and Figure 2).

## 4. BLV Susceptibility

Generally, retroviruses have a wide host range that includes cattle, buffaloes, sheep, and goats [113,114]. Three species are natural hosts of BLV infection:, *Bos taurus* (domestic cattle), *Bos indicus* (zebu), and *Bubalus bubalis* (water buffalo) [15]. The disease is not confirmed in some susceptible species (e.g., capybara, rhesus monkeys, chimpanzees, and antelopes), and has not been detected in other wildlife species, such as deer and llama, under natural conditions [115,116]. Experimentally, sheep, goats, pigs, chickens, rats, and rabbits have been infected [117], but the disease has a shorter period of latency, takes over the animal more quickly and is passed on more frequently than in cattle. Rats are one of the suitable models for in vivo studies of BLV infection [118].

Estimates of bovine leucosis inheritability among Holstein and Jersey cattle populations is about 0.08% [119], which indicates that genetic susceptibility plays a role in the prevalence of BLV in some breeds of cattle [120]. BLV is more frequently spread among dairy than among beef cattle [121]. The intra-herd seroprevalence rates in dairy and beef cattle are reported to be 40.9% and 28.7%, respectively [102,122]. In China, 49.1% of dairy and 1.6% of beef cattle have been found to be BLV-positive [99]. Furthermore, the age of cattle plays a pivotal role in the seroprevalence of BLV, which increases with age until cattle older than two years show seroprevalence rates that are almost twice those found in younger animals [123]. This is because the risk of infection increases as animals spend more time at risk of contact with it [123]. However, EBL has been detected in younger animals; in a two-month-old calf [124], in two calves of around three months of age [125] and in a 13-month-old Holstein heifer [123]. Therefore, more consideration should be given to the possibility of BLV infection regardless of age.

## 5. Transmission

BLV has a harsh transmission dynamic, as the virus exists in circulating peripheral blood lymphocytes of infected animals and the disease can be transmitted by both horizontal and vertical means [126,127,128]. The critical sources of BLV infection are fresh blood, semen, saliva, milk, and nasal discharges of BLV-seropositive cattle that have PL and that harbour proviral DNA (which enables cell-to-cell transmission) [1,39,127,129,130].

Horizontal or mechanical transmission plays a major role in BLV infection via several potential routes that include all practices performed beyond blood transmission control, such as iatrogenic infection during vaccination, blood extraction, castration, injection of medication, dehorning, tattooing, and rectal palpation [131,132,133]. Another significant risk for horizontal BLV dissemination is posed by biting flies, such as the stable fly, which can carry the virus from the infected blood of a host animal to a susceptible other during a blood meal and pose a potential risk of infection [40,134]. The crucial role of biting flies in the epidemiology and prevalence of BLV has been identified by two epidemiological studies that were performed in the US and Japan [81]. It was reported that no new cases of EBL were observed in Japanese beef cattle herds after herdsmen implemented rigorous fly control [135,136,137]. This study showed that passage was via a blood-borne pathogen, such as HTLV-1 or HTLV-2 [1].

A small proportion of BLV infections may occur vertically via the maternal-foetal transmission route from the dam to its foetus or through the ingestion of colostrum and milk that contains provirus or free virus particles. Such transmission has been positively correlated to the proviral loads that have been measured in the dam [138]. Milk cells from BLV-infected cows could cause infection *ex vivo*, which suggested a potential risk from milk for vertical spread of BLV via cell-to-cell transmission [127]. However, a high antibody titre in the milk and colostrum of BLV-positive dams could protect against BLV infection in vitro [139]. The in utero or transplacental transmission also shares a route of vertical BLV transfer from the dam to her offspring [140]. Therefore, the BLV proviral load and antibody titre in the milk play a direct role in the infection and protection of calves against the disease.

Artificial insemination (AI) and natural service might also be incriminated in the vertical spread of BLV. Although the risk of in vitro infection with semen from BLV-infected bulls is negligible, the use of infected bulls in natural service has been positively associated with BLV prevalence [141]. Moreover, during natural copulation, the smegma of infected bulls may aid in the transmission of BLV to uninfected cows. So, bulls that are used for natural mating or AI must be screened to ensure that they are BLV-free before the breeding season to aid in the limitation of virus spread [142].

## 6. BLV Pathogenesis

BLV can infect different immune cells that show the highest preferential affinity to B-lymphocytes. It presents in the circulating peripheral blood B lymphocytes of BLV-infected cattle and less often in T-cells [143,144]. It disrupts both B- and T-cell homeostasis and alters their proliferative and apoptotic responses as it interferes with gene expression and the actions of signalling cascades at different times post-infection [10]. Cows that develop PL undergo a massive proliferation of B-lymphocytes that express both Ig and CD5^+^ antigens on their surface via blockage of their apoptosis rather than triggering of their proliferation [145]. The BLV structural genes *pol* and *env* are essential for in vivo infectivity and their deletion eliminates infectivity. Moreover, the polymorphism of the *env* gene leads to a change in viral pathogenicity [146].

Inactivation of tumour suppressor gene p53 by mutation appears to be one of the critical events that is associated with tumour formation by BLV in cattle but not in sheep [147]. Additionally, sheep show a low level of apoptosis when compared with cattle, because sheep are not natural hosts of BLV; protection against apoptosis at the early asymptomatic stages of the disease seems to result in slower development of leukaemia and reduced pathogenicity [39].

During infection by BLV, a transmembrane glycoprotein of gp51 destabilises the host cell membrane with a fusion peptide, after which the structural proteins enhance viral fusion and infectivity of host cells [148]. Further, it has been noted that mutation of a single envelope N-linked glycosylation site (N 230 E) of the *env* gene by conversion of the asparagine codon (N) into glutamic acid (E) enhances the pathogenicity of BLV through enhancement of viral replication, fusogenicity, and protein stability in experimentally infected sheep [41].

After virus entry, there is no detectable viraemia, but there is a strong and persistent humoural immune response to structural proteins, especially against the env gp51 and the major core protein p24 [149]. Synthesis of BLV proviral DNA molecules is achieved by viral reverse transcriptase. Then, with the help of viral integrase, the provirus is inserted at random sites into the host genome in the nucleus of the infected cells [150]. The BLV provirus remains integrated into cellular genomes for life, even in the absence of detectable BLV antibodies, and viral transcription is blocked during the latent period of the disease which is called the silent state [106]. It has been recorded that, when an infected cell with an integrated BLV provirus is transmitted into a new host, the BLV provirus is expressed into viral particles that infect other B lymphocytes [151].

The BLV epitopes have an important influence on the viral life cycle. BLV has three conformational and neutralising epitopes named F, G, and H, which are always found among all BLV strains. This suggests that their presence is essential for viral pathogenesis. Antibodies that are synthesised against epitope H can prevent cell fusion in culture, while those against epitopes F and G only can reduce the syncytial ability of the virus [152]. Regarding the occurrence of B-cell lymphosarcoma, BLV encodes some miRNAs that are essential for the induction of B-cell tumours and they regulate efficient viral replication in vivo [71]. In this area, the pathogenesis of BLV infection is not fully clear and requires further investigation.

## 7. Clinical Outcomes

Generally, bovine leucosis takes two forms: the first is a fatal EBL form that is characterised by lymphomas; the second is sporadic bovine leucosis (SBL), which is not transmissible and mainly affects young calves [153]. Furthermore, EBL can be separated into three forms as follows: first, the asymptomatic form, which shows the most frequent occurrence (70%) and infected animals appear to be serologically positive without lymphocytosis or any clinical signs; second, the form that causes animals to be serologically positive and positive for PL (30%). This form causes a non-malignant polyclonal expansion of CD5^+^ B cells, the majority of which harbour the BLV provirus with high viral load; and the final form exhibits as malignant lymphosarcoma (less than 5%). It originates from mono or polyclonal accumulation of CD5^+^ IgM^+^ B cells after a long period of latency that may extend to one to eight years, so it is detected at a higher rate in cattle of more than four to five years old rather than in younger animals [58,154,155,156]. Thus, persistent B-lymphocyte proliferation is the hallmark of BLV-induced leucosis and is referred to as leukaemia in the bloodstream, lymphoma in the lymph node and lymphosarcoma in various organs [147].

The clinical picture of the disease may include the following manifestations: lack of appetite, indigestion, reduced milk yield, chronic bloat, displaced abomasum, diarrhoea, constipation, enlargement of superficial lymph nodes, lameness, paralysis, weight loss, weakness or general debilitation, and sometimes neurological manifestations [106,157].

A field study on BLV-infected cattle in Egypt reported various symptoms, such as lymph node enlargement (6.25%), protrusion of conjunctival membrane (1.67%), lameness (0.42%), emaciation (1.25%), and respiratory manifestations as rales and dry cough (0.83%) [80]. Cattle with lymphosarcoma almost invariably die, either suddenly or weeks or months after the onset of clinical signs, which differ according to the particular organ(s) involved [158]. The BLV malignancies disrupt the uterus, mesenteric, retro-bulbar, the right auricle of the heart, abomasum, spleen, lung, kidney, urinary tract, spine, and pre-scapular, and sub-iliac lymph nodes [151]. These disruptions result in urinary, respiratory, and digestive disturbances besides other signs according to the organ involved [58].

Recently, it has been speculated that BLV infection decreases the energy production efficiency of cows as it alters the activities of their ruminal and intestinal microbiota, which rely partially on the multiplication ability of BLV strains [159]. Additionally, BLV causes dysfunction of monocytes and neutrophils, which subsequently leads to immunosuppression. Both these effects may explain the elevated susceptibility of the animals to other infections, their reduced milk production and reproductive inefficiency [160,161].

Immunologically, the mechanisms by which BLV immunity is compromised are not clear but several scenarios have been suggested, including (1) disruption of cytokine production and correct immune cell signalling, (2) proliferative and apoptotic disturbance of immune cells, and (3) possible damage caused by activated and infected cells. Therefore, future research on BLV immunology and on how BLV influences response to vaccines and other pathogens is urgently required to calculate the exact economic impact of BLV infection on the cattle industry [10].

## 8. Zoonotic Potential of BLV

The presence of BLV or BLV-infected cells in the milk of most naturally affected cows suggests that humans are frequently exposed to BLV orally [162]. The first indication of BLV’s potential impact on public health came from a study conducted in the 1970s, just a few years after the virus was discovered, in which two out of every six chimpanzees fed unpasteurised milk from naturally infected BLV cows developed fatal erythroleukemia [163]. Extensive epidemiological investigations conducted in the United States, Denmark, and Sweden failed to show a link between human leukaemia and bovine leukaemia [164]. Anti-BLV antibodies were not detected in persons with varying levels of BLV exposure in seroepidemiological studies. The lack of BLV-specific sequences in 157 cases of childhood acute lymphoblastic leukaemia or non-lymphoma Hodgkin’s and 136 controls in the United States [165], as well as 517 cases of human leukaemia and 162 lung cancer patients in Korea [166], provided additional evidence against BLV’s involvement in human disease.

Despite the fact that research in the past have revealed a link between BLV and breast cancer, the evidence remains inconclusive. By sensitive enzyme-linked immunosorbent test or PCR, no anti-BLV antibodies or BLV sequences were detected in Chinese healthy or breast cancer patients [167].

Recent studies using more sensitive whole genome sequencing, have found no indication of a link between BLV and human breast cancer. None of the 32 billion sequencing reads collected from 51 breast tumours matched BLV strains [168].

## 9. Diagnosis

To improve recognition of BLV infection and to control its spread, efficient diagnostic techniques should be established for routine and easy application in order to pick out the infected animals. This is why different serological tests are widely used to screen animals for BLV, as shown in Table 3. Although serological tests can provide rapid screening, they are less sensitive than others mentioned earlier and they cannot be adapted for tissue or semen testing [169]. Furthermore, they provide no information regarding the BLV proviral load and the degree of BLV-induced disease progression [170]. They also cannot be used to detect low and transient levels of BLV infection, for which a PCR test can be used (Table 3 and Table 4).

Among serological tests, ELISA and AGID are the reference techniques that are recommended by the OIE for diagnosis of BLV infection through the detection of antibodies that are directed to BLV gp51 and p24 proteins. Although AGID is the gold standard, ELISAs have been frequently used due to their higher sensitivity [39,78,185,186]. Moreover, in one study, the AGID test failed to detect the infection in up to 30% of animals that were found by other methods to be positive for BLV [187] and failed to detect BLV-infected animals at a large scale from pooled serum or milk samples, whereas some commercially available ELISAs have been found effective in such cases [188,189] with varying degrees of sensitivity (between 97% and 100%) and specificity (between 78% and 100%) [186,190].

Compared with serological assays, the development of highly sensitive and more specific molecular techniques, especially those based on different kinds of PCR, has revolutionised the diagnosis of BLV and other viral diseases [191]. So, the detection of BLV proviral DNA is a useful tool to discover whether an animal is BLV-infected or not [192].

PCR tests can detect directly the presence of proviral DNA in BLV-infected cattle that have low, transient, or absent antibody titres. After performance of the PCR, sequencing and phylogenetic analysis enable the study of the distribution of BLV genotypes worldwide. Additionally, a PCR test can be used to differentiate lymphomas that are induced by BLV from those associated with SBL [193]. However, it may fail to detect some cases that are seropositive on ELISA due to the presence of extremely small amounts of provirus genetic material in the lymphocytes of infected animals [194], infection confined to lymphoid tissues rather than circulating lymphocytes [195], or the presence of *taq* DNA polymerase inhibitors in DNA samples [192].

There are different types of PCR that are useful for different purposes. Each of the conventional single, semi-nested, and nested PCR tests is a useful and sensitive tool that can be used to detect BLV proviral DNA early in blood, organs, or tumour samples, but the semi-nested and nested tests provide much higher levels of sensitivity than single PCR, as is summarised in Table 3.

Performance of a single PCR followed by sequencing and phylogenetic analysis of the BLV *env*, *gag*, or *pol* genes, with the interpretation of genotypic clusters of the constructed dendrograms, is the most efficient approach for BLV gene identification and reveals the BLV genetic variations that are found worldwide [89,196]. A nested PCR is a specific and reliable method that has various uses: to detect BLV in young calves that have been fed with colostrum from seropositive cows; to differentiate between sporadic and infectious lymphomas (enzootic) in tumour tissues gathered from suspected cases in slaughterhouses, in recent infections before the development of antibodies; as a check when ELISA results are doubtful or show weak positive reactions; and for surveillance of bulls in progeny tests before they are used in AI centres [86].

Another rapid and simple type of PCR is the direct, blood-based PCR system (PCR-DB), which was developed to amplify the amount BLV provirus directly from whole blood without DNA extraction and purification. This method is of lower sensitivity than nested PCR but indicates higher specificity and reproducibility, and is cost-effective as it is neither labour-intensive nor time-consuming [11]. Further, a direct filter PCR test has been established recently as a novel, fast, smooth, reliable, and practical diagnostic test that directly detects the BLV proviral DNA in clinical blood samples without DNA extraction while offering simple collection, transportation and storage procedures for clinical blood specimens [184].

Since the proviral load of BLV plays a significant role in both disease progress and prognosis, the need for some molecular techniques to quantify the viral copies in an infected animal became imperative in both diagnosis and eradication strategies of bovine leucosis [182]. A quantitative, real-time PCR for BLV that is based on the SYBR family of dyes is a confirmatory method that shows high sensitivity in the detection of BLV proviral load in infected cattle with low, transient, or undetectable antibody levels during the early stage of infection. Its use is recommended to elucidate the BLV disease status of animals that show uncertain ELISA results in tests of their serum [136,197,198,199,200].

A quantitative PCR named the BLV coordination of common motifs qPCR (BLVCoCoMo-qPCR) is another highly specific and sensitive diagnostic technique that can be used to detect BLV in specimens that showed negative results in nested PCR tests. It detects various integrated BLV strains within the host genome in clinical cases from a broad geographical origin [170]. This technique can also be used to measure the BLV proviral load of both known and novel BLV variants. Hence, it can be used subsequently to demonstrate the correlation between BLV proviral load and disease progression [201,202].

Luminescence syncytium induction assay (LuSIA) is an easy, highly sensitive and rapid method for identification and quantification of BLV infection. Therefore, it may be effective for high-throughput screening of several samples or for prolonged follow-up surveys. It may also be useful in the detection of BLV-specific antibodies, validation of BLV vaccine candidates, and detection of chemical compounds that are used to treat BLV-infected animals. Thus, LuSIA may be highly beneficial for diagnosis and in the quest to suppress the horizontal spread of BLV [203]. A new, more sensitive, and quantitative protocol for LuSIA to measure BLV infectivity has been established by use of CC81-BLU3G, CC81-GREMG, and CC81-GREMG-CAT1 cells. It is adaptable to several assays including: BLV neutralisation by plasma or serum; screening of anti-viral drugs; and BLV contamination contradiction assay of bovine vaccines. It also can be used to detect both cell-to-cell and cell-free infection of BLV sensitively and at an early time point [204,205].

BLV isolation in cell culture is also an efficient way to detect BLV infection and to study both its viral biology and life cycle. BLV infects a wide range of cell lines derived from various mammalian species and organs; however, the production of viral progeny has been shown to be restricted to some cell lines only [206]. In vitro cultivation of infected polymorph nuclear cells (PBMCs) on foetal bovine lung cells (FBL) or Madin–Darby bovine kidney (MDBK) cells leads to viral infection and replication with the production of cytopathic effects (CPE) in a form formation of cell syncytia [207,208].

In addition to the previously mentioned approaches for BLV diagnosis, changes in animals’ haemato-biochemical and oxidative status may serve as an indicator of BLV infection. Infection with BLV is associated with a selective reduction in glutathione peroxidase activity without any change in levels of the common plasma oxidative stress markers (i.e., hydroperoxides, conjugated dienes, and malondialdehyde) [209]. The leukaemic lymphocytic cellular infiltration in hepatic and renal tissues promotes disorders of liver and kidney function and impairment of nephron filtration ability because of nephron damage that appears in the early stage of leukosis [210,211]. Therefore, the infection is associated with increased activity of the liver enzymes alanine transaminase, alkaline phosphatase, and aspartate transaminase, and of creatinine and superoxide dismutase. On the other hand, the calcium level is significantly decreased and non-significant alterations have been recorded in levels of malondialdehyde and nitric oxide [212]. BLV infection also significantly affects the haematological parameters of infected animals; BLV-seropositive cattle show significant increases in counts of lymphocytes, leukocytes, monocytes, and neutrophils when compared with seronegative animals. This finding may support the hypothesis that BLV can affect host immune response [213].

## 10. Strategies for the Control of BLV Infection

Programmes to eradicate BLV infection are considered a worldwide challenge. They have never been considered economically feasible, especially when the disease prevalence is high [14,214]. Several attempts have been made to control BLV infection and to decrease the disease frequency in the herds, especially for multiparous cows with ≥5 parities that live on large farms with more than 200 cows, since these are considered to be among the main risk factors [112].

Four main strategies can be followed to prevent BLV infection: test and cull, genetic selection, good management practice, and vaccination. Several European regions where BLV prevalence is low successfully implement a policy to test all animals and cull those that are found to be positive alongside ongoing surveillance for the evidence of disease [18]. Regions with high prevalence rates should reduce these rates to a point at which test and cull is economically feasible [18]. This strategy involves periodic screening through use of ELISA, PCR-DB, and real-time PCR followed by segregation of all positive cases and of animals that exhibit high proviral load (more than three BLV copies/100 cells) at intervals of several months or a few years [11,88,182]. Additionally, the culling of older cows with high whole blood and lymphocyte counts helps to control BLV infection and is considered the cheapest screening method as it avoids the need for further testing [215,216].

Eradication of BLV infection could also be achieved through genetic selection of animals that carry resistance genes in their major histocompatibility complex class II molecules, because cattle with the BoLA class II DRB3 * 0902 allele have been found to be BLV-resistant or to show significantly low levels of BLV proviral load. However, such a strategy could have critical drawbacks regarding the susceptibility of the genetically selected animals to other fatal diseases in the future [2,217].

Disease prevalence can also be reduced by application of safety procedures, such as introduction of an appropriate quarantine period when cattle arrive on a farm and serological testing against BLV antibodies alongside use of a closed trading system that avoids the introduction of animals from infected localities. Focus on the between-farm movement of animals is crucial [218]. Traditional management practices are recommended for the control of BLV transmission, including: the single use of hypodermic needles and reproductive examination sleeves; use of AI instead of natural breeding; control of biting arthropods; feeding calves only heat-treated colostrum or colostrum replacements; and the cleaning and disinfection of blood-contaminated equipment that is reused during surgical operations, such as application of ear tags, tattooing, and dehorning through use of chloroform, ether, and UV. Application of all these practices might eventually reduce the prevalence of BLV-infected cattle to a sufficiently low level to introduce a test and cull policy [39,219]. Moreover, vertical disease spread can be avoided by the freezing and thawing of the colostrum and milk (at −25 °C for one night followed by thawing) before feeding calves. This system could be used by veterinarians and farmers in the development of an effective BLV control programme [220].

As with other viral diseases, a vaccination programme is urgently required to control BLV infection, and a vaccine against bovine leucosis is in great demand. However, individual differences in sensitivity to the disease make it difficult to assess the efficiency of a candidate vaccine [221]. Several epitopes have been obtained from gp51, gp30, and Tax, and this work has demonstrated that these proteins are heavily involved in development of cellular immunity. The gp51p16-C and CD8+CTL epitopes from gp30 and Tax proteins are particularly beneficial to provide a potent target for BLV monoclonal antibody production. They may greatly facilitate the development of therapeutic and prophylactic strategies for BLV [222,223]. Efficient, safe BLV vaccine has been produced through use of recombinant vaccines that are vectored by lumpy skin disease virus (LSDV) against both BLV and LSDV. Expression of the BLV *env* and *gag* antigens from the recombinant vaccine was confirmed [224].

Despite trials of vaccines against BLV, there is no effective therapy or commercial vaccine yet available for the control of EBL, mainly because trials show only an incomplete or transient stimulation of the host immune response [11,225].

## 11. Conclusions

Bovine leucosis is an important disease that affects the economy of localities in which it is endemic through its withering effect on the animal, either directly or indirectly. The most successful means for its eradication is the testing and culling of infected animals with the implementation of a closed trading system that prevents the introduction of new animals from infected areas. Such a system is followed by most European countries. Control of EBL at the national level usually involves one or more of the following four approaches: test and segregation or test and slaughter; genetic selection; management interventions; and development of a novel efficient vaccine.

## 12. Future Prospective Studies

Based on the previously reported data, the need for an innovative BLV vaccine has become critical. Therefore, research should be directed toward the development of a novel marker BLV vaccine that omits single or multiple genes related to viral immunosuppression or virulence. Introduction of such a vaccine would reduce BLV prevalence rates worldwide to controllable levels. Then, a test and cull strategy should be implemented. Both together may offer a way to eradicate the disease at the global level. Furthermore, BLV proteomic analysis should be considered, as knowledge of the protein composition of BLV would increase understanding of important characteristics, such as how the virus interacts with host cells. Such understanding would subsequently lead to valuable knowledge for the elucidation of viral biologies, such as replication, tropism, virulence, and immunogenicity.

## Figures and Tables

**Table 1:** BLV Genotyping based on partial BLV env sequences identified in geographical locations around the world.

Continent	Countries	Genotype	References
Europe	France	4	[44,50,53,58,59,60,61,62,63,64]
	Belgium	4	
	Moldova	7	
Asia	Korea	1&3	
	Japan	1&2&3	
	Russia	4&7&8	
	Thailand	1&6&10	
	Myanmar	1&6&10	
	China	4&6&11	
	Philippines	1&6	
	Iran	4	
Australia	Australia	1	
North America	USA	1&3	
Central America	Costa Rica	1&5	
South America	Brazil	1&2&6	
	Uruguay	1	
	Paraguay	1&6	
	Bolivia	9	
	Argentine	1&2&4&6	
	Peru	2	
	Colombia	1&3&6	
Africa	Egypt	1&4	
	South Africa	1&4	
	Zambia	1	

**Table 2:** Prevalence of the bovine leucosis disease worldwide, adopted from [58].

Status	Continent	Countries	Year	References
BLV free countries	Europe	Andorra	1994	[85]
		Cyprus	1995	[85]
		Czech Republic	2010	[85]
		Finland	2008	[85]
		Ireland	1999	[85]
		Norway	2002	[85]
		Spain	1994	[85]
		UK	1996	[85]
		The Netherlands	2009	[86]
		Sweden	2007	[85]
		Denmark	1990	[85]
		Estonia	2013	[85]
		Switzerland	2005	[85]
		Slovenia	2006	[85]
	Oceania	Australia	2013	[12]
		New Zealand	2008	[87]
		Tunisia	2005	[85]
	Asia	Kyrgyzstan	2008	[85]
		Kazakhstan	2007	[85]
BLV existing countries with unknown prevalence	Europe	Croatia	Present	[86,88]
		Ukraine		[89]
		Italy		[85]
		Portugal		[85]
		Belarus		[86,89]
		Greece		[85]
		Bulgaria		[85]
		Latvia		[85]
	South America	Uruguay		[90]
BLV existing countries with variable prevalence	North America	USA (Dairy 83.9%, Beef 39%)	2007	[91]
		Mexico (Dairy 36.1%, Beef 4%)	1983	[92]
		Canada (78% at herd level)	1998–2003	[5]
	South America	Chile (southern regions) (27.9% individual level)	2009	[48]
		Brazil 17.1% 60.8%	1980–1989 1992–1995	[93,94]
		Argentina (Buenos Aires) (77.4% at an individual, 90.9% at herd level) (Multiple regions) (32.85% at individual, 84% at herd level)	2007 1998–1999	[48] [95]
		Peru (Multiple regions) (31% at the individual level, 42.3% individual level)	1983 2008	[96] [48]
		Bolivia (Multiple regions) 30.7% individual level	2008	[48]
		Venezuela 33.3% individual level	1978	[97]
		Paragua (Asuncion) 54.7% individual level	2008	[48]
		Colombia 62% individual level	2020	[98]
	Asia	China (Dairy 49.1%, Beef 1.6%)	2013–2014	[99]
		Taiwan (81.8% at animal level and 99.1% at herd level)	2019	[100]
		Cambodia Draught cattle 5.3%	2000	[101]
		Japan (Nationwide) Dairy 49.1% Beef 1.6% 79.1% of the dairy herd 73.3% at individual	2009–2011 2007 2012–2014	[102] [103] [104]
		Mongolia (Dairy 3.9%)	2014	[105]
		Iran (nationwide) (22.1% to 25.4%)	2012–2014	[5]
		Philippines (4.8% to 9.7%)	2010–2012	[106]
		Myanmar (9.1% at individual)	2016	[48]
		Thailand (58.7% at individual)	2013–2014	[53]
		Pakistan (20% of dairy)	2019	[107]
	Middle East	Saudi Arabia (20.2% of dairy)	1990	[108]
		Turkey (48.3% dairy)		[109]
		Israel (5% at individual)		[110]
		Iraq (7% of dairy)	2015	[111]
		Egypt (17.7% of dairy)	2020	[112]

**Table 3:** Serological techniques used for diagnosis of BLV prevalence according to the rewarded samples and test sensitivity, reproduced from [58].

Rewarded Samples	Test	Advantages	Disadvantages	References
Serum sample	1. ELISA (Antibodies p24, gp51)	Sensitive, specific, large scale screening and rapid	False negatives (cattle in the early infection phase) False-positive (maternally derived antibodies) Cannot evaluate disease states of infected cattle.	[157,170,171,172,173]
	2. RIA (Antibodies p24)	Sensitive Able to detect BLV during the early period of infection	Cannot be used for mass screening	[174,175]
	3. AGIDT (Antibodies p24, gp51)	Specific, simple, rapid, screening and Less expensive	Less sensitive Inconclusive Fail to evaluate disease states	[9,171,172,173]
Milk and Bulk milk sample	ELISA [157,170,171,172,173]			
Virus particle	PHA (BLV glycoprotein)	Sensitive, Specific, Less expensive, and Rapid	Affected by pH and temperature Hemagglutination activity reduced by trypsin and neuraminidase	[176]

**Table 4:** Molecular techniques used for diagnosis of BLV prevalence according to the rewarded samples and test sensitivity (All detect proviral DNA), adopted from [58].

Rewarded Samples	Test	Advantages	Disadvantages	References
(Blood, PBMC, Tumour sample, Buffy coat, Milk, somatic cells, Semen, Saliva and Nasal secretions).	Realtime PCR	Direct, fast, sensitive, and Low risk of contamination. Differentiate EBL from SBL. Detect BLV during the early phase of infection or in the presence of colostrum antibodies. Can detect BLV proviral load.	Needs complicated sample preparation Requires specific primers and probes Expensive and Require equipment (real-time PCR machine)	[170,177,178,179]
	Conventional PCR (Single, Semi-Nested, and Nested PCR)	Direct, fast, sensitive and can detect recent infections, before the development of antibodies to BLV. Can be used BLV detection during the early phase of infection or in the presence of colostrum antibodies.	False-negative in case of low proviral load and the presence of PCR inhibitory substances in samples Ease of cross-contamination Requires specific primers Requires equipment (PCR machine) Needs sequencing for confirmation	[157,171,173,180,181]
Blood only	Direct blood-based PCR	Not expensive Applied on the blood directly without DNA extraction nor purification Low risk of contamination	False-negative in case of low proviral load less sensitivity	[11,182,183]
Blood only	Direct filter PCR	Novel, rapid, easy, reliable, and cost-effective diagnostic test No need for DNA extraction Offers simple collection, transportation, and storage procedures for clinical blood specimens	False-negative in case of low proviral load less sensitivity	[184]

## Data Availability

Data sharing not applicable.
